# Efficacy of hyperthermic isolated limb perfusion in the treatment of locally recurrent high-grade soft tissue sarcoma of the extremities

**DOI:** 10.1186/s12957-020-02110-4

**Published:** 2020-12-21

**Authors:** Farhad Farzaliyev, Alexander Touma, Georg Taeger, Hans-Ulrich Steinau, Jendrik Hardes, Arne Streitbürger, Lars Erik Podleska

**Affiliations:** 1 Department of General, Visceral and Transplantation Surgery, Division of Plastic and Reconstructive Surgery, University Hospital Essen, University Essen-Duisburg, Hufelandstr.55, 45147 Essen, Germany; 2 Department of Tumor Orthopedics and Sarcoma Surgery, University Hospital Essen, Essen, Germany

## Abstract

**Background:**

This novel study compared the use of tumor necrosis factor (TNF)-alpha and melphalan-based isolated limb perfusion (TM-ILP) to the standard treatment of locally recurrent soft tissue extremity sarcoma. The aim was to assess whether TM-ILP positively influences the recurrence-free survival of locally recurrent high-grade soft tissue sarcoma (STS) of the extremities.

**Methods:**

We retrospectively analyzed our clinical database for patients with STS. Variables were analyzed using chi-square test or Mann-Whitney rank-sum test. Furthermore, Kaplan-Meier survival plots were calculated and a proportional hazard regression model was developed.

**Results:**

Out of 448 patients with extraabdominal STS treated between August 2012 and December 2015, 52 cases involving 47 patients had locally recurrent STS. Twenty-eight of these patients were treated with TM-ILP prior to surgical resection (TM-ILP-group), and 24 were treated with standard therapy (without TM-ILP). The 3-year recurrence-free survival for the TM-ILP-group was estimated at 75% (95% confidence interval (CI), 71.5–78.5). Local recurrence-free survival in the standard group was significantly lower (LRFS: 43.4%, 95% CI 38.7–48.1, *p* = 0.026). Multivariable analysis revealed resection with negative margins, lower number of previous recurrences, and TM-ILP as positive predictors for recurrence-free survival.

**Conclusions:**

TM-ILP and consecutive resection of residual tumor with negative resection margins significantly improves local recurrence-free survival for patients with a first local recurrence of high-grade STS in the extremities.

## Background

Soft tissue sarcomas (STS) represent a mixed group of malignancies that are characterized by their mesodermal origin. The most common location of STS is found in the extremities (59%) [1]. Resection of the tumor with adequate safety margins remains standard treatment whenever possible. High-grade STS can grow asymptomatically as a painless lump; therefore, deep-seated STSs often tend to be large, locally advanced tumors at time of diagnosis. In tumors that are considered non-resectable—meaning that adequate safety margins are impossible without amputation or severe mutilation—a multimodal approach is currently considered standard treatment [2]. Management of local recurrences is often difficult due to the surgically altered anatomy of these tumors as well as dose limitations because of previously administered radiation therapy [3].

Hyperthermic isolated limb perfusion with TNF-alpha and melphalan (TM-ILP) is a useful tool in a multidisciplinary treatment regime and is increasingly implemented for the treatment of locally advanced STS in otherwise unsalvageable limbs. ILP is often used in a primarily curative intention, if applicable. In these cases, the subsequent resection of the devitalized and residual tumor is an integral part of the treatment concept. Response rates of TM-ILP are reported to range between 60 and 80%, resulting in limb salvage rates above 80% [4–7].

Previous studies revealed that local recurrence rates were not higher in primary advanced STS tumors treated with TM-ILP and subsequent resection of the residual tumor than those treated by a combination of radiation therapy and surgery [8]. Presently, there is no published data regarding the efficacy of TM-ILP in locally recurrent extremity STS. All current studies regarding TM-ILP are derived from pooled data of primary and locally recurrent tumors [9–11].

The aim of this study was to compare the oncological outcome parameters (manifestation of local recurrence or distant metastasis) of patients treated for recurrent high-grade soft tissue sarcoma of the extremities with a combination of TM-ILP and subsequent tumor-resection to a group of patients treated with standard therapy (tumor resection without TM-ILP).

## Patients and methods

### Patients and data collection

This retrospective study included 448 patients with extraabdominal STS treated between August 2012 and December 2015. Analysis of this data revealed 52 cases from 47 patients who had a histologically confirmed locally recurrent high-grade STS of the extremities (excluding tumors extending into or beyond the axilla and groin).

Contrast medium enhanced MRI was used for local staging of the tumor prior to TM-ILP (if applicable) and prior to tumor resection. Computed tomography of the chest and abdomen was performed for systemic staging. Resectability of recurrences was estimated in an interdisciplinary tumor board consisting of specialists from surgical oncology, medical oncology, radiation therapy, radiology, and pathology.

TM-ILP was performed 6 weeks prior to resection of the residual tumor in a standardized manner as previously described [12].

All tumor resections were performed at our university hospital by the same team of three experienced sarcoma surgeons as defined by Tang 2009 [13]. Resections were planned with tumor-negative margins. Whenever possible, tumor resections were performed with adequate safety margins.

### Pathology

Histopathological assessment of the resection specimen was performed in a standardized manner; tumors were transected in slices of 10 mm. Resection margins and treatment response (if applicable) were first estimated from gross pathology. Areas of special interest (closest margins) were marked with ink, and a minimum of one block per centimeter of tumor diameter was embedded for further microscopic analysis. Final evaluation of resection margins and treatment response (as percent of necrotic tumor tissue) was performed on H&E-stained slides. Typing and grading of tumors were determined according to WHO and TNM classifications [14]. Pathological complete response (CR) was defined as the nonexistence of identifiable residual tumor cells, a very good response (GR), between 1 and 10% of recognizable tumor cells, a partial response (PR), the presence of between 11 and 50% of recognizable tumors cells, and no change (NC) if > 50% of recognizable tumor cells were present in tumor specimen [10].

### Collection of data and follow-up data

Patients were either treated by TM-ILP and subsequent tumor resection (*TM-ILP-group*), or tumor resection alone (*non-TM-ILP-group/standard group*).

Follow-up data from patients was collected during their regular follow-up examinations according to the NCCN-Guidelines for STS (www.nccn.org/professionals/physician_gls #soft-tissue-sarcoma) in 3- to 6-month intervals [2].

### Statistical analysis

Chi-squared test was used to compare categorical variables. If expected cell frequencies were below five, a Monte-Carlo simulation (500 subjects, 95%) was performed. For continuous variables, the Mann-Whitney rank-sum test was employed.

Failure of local therapy, resulting in a subsequent local recurrence, was defined as relapse of the tumor within the operated area more than 3 months after surgery. Metastasis was defined as identification of primary tumor histology in any other location. Primary endpoint was subsequent recurrence-free survival (SRFS), measured from date of local recurrence resection to time of subsequent local recurrence, or date of last follow-up in the absence of subsequent local recurrence. Secondary endpoint was additional subsequent recurrence free survival, which was defined as time to local relapse after treatment of subsequent local recurrence. Tertiary endpoint was distant metastasis-free survival, measured from the date of surgery for local recurrence to time of first distant relapse or to date of the last follow-up if no distant relapse occurred. Periods at risk of new local recurrence and metastasis were defined in months for each patient. Patients with existing and successfully treated metastasis prior to relapse were censored. If, during the study period, a local relapse was diagnosed more than 3 months after surgical removal of a previous recurrence, this patient was identified as a new case for SRFS. For univariable analyses, Kaplan-Meier and log-rank test were performed. Multivariable analysis employed Cox regression models with forward and backward stepwise selection (inclusion criterion: *p* value of the score test < 0.05, exclusion criterion: *p* value of the likelihood ratio test > 0.10). The following variables were included for the Cox regression models of SRFS: age, sex, tumor location and size, histology, radiation therapy of the previous tumor, resectability, resection margins, TM-ILP, number of recurrences, and current radiation therapy.

Statistical analysis was performed with SPSS (Statistical Package for the Social Sciences) software, version 23.0.

## Results

Out of 52 cases, 27 (52%) were referred to our clinic after external therapy of the primary tumors, 28 cases (54%) underwent surgery after TM-ILP (TM-ILP-group), and 24 (46%) were treated by surgery alone (standard group). There was no statistically significant difference between the two groups in terms of tumor localization (*p* = 0.101), tumor size (*p* = 0.154), histological subtypes (*p* = 0.146), and previous radiation therapy for the primary sarcomas (*p* = 0.966). TM-ILP was performed more often in female patients than in male patients (34.5% vs. 19%; *p* = 0.026) (Table 1). Pathological CR after TM-ILP was in three patients (10.3%), GR in four (13.8%), PR in eight (27.6%), and NC in 14 (48.3%).

**Table 1:** Patient demographics, tumor, and previous therapy characteristics

	Standard group (*n* = 24)	TM-ILP group (*n* = 28)
Median age (mean and range)	63 (32–85)	58 (25–74)
Sex		
Male	16 (31%)	10 (19%)
Female	8 (15%)	18 (35%)
Localization		
Upper arm	2 (4%)	4 (8%)
Lower arm and hand	2 (4%)	4 (8%)
Upper leg	15 (29%)	9 (17%)
Lower leg and foot	5 (10%)	11 (21%)
Recurrences		
First recurrence	11 (21%)	15 (29%)
Second and additional recurrences	13 (25%)	13 (25%)
Histopathology		
Liposarcoma	7 (14%)	4 (8%)
Undifferentiated soft tissue sarcoma	7 (14%)	11 (21%)
(Myxo-)/fibrosarcoma	5 (10%)	2 (4%)
Synovial sarcoma	0 (0%)	4 (8%)
Others	5 (10%)	7 (14%)
Tumor size		
T1	3 (6%)	10 (19%)
T2	16 (31%)	15 (29%)
> T3	5 (10%)	3 (6%)
Previous radiation therapy		
Yes	13 (25%)	15 (29%)
No	11 (21%)	13 (25%)
Previous surgery		
In domo	15 (29%)	10 (19%)
Ex domo	9 (17%)	18 (35%)

Although patients treated with TM-ILP had significantly more non-resectable tumors (50% vs. 29%, *p* = 0.008), and there was no statistically significant difference in terms of resection margins (*p* = 0.898), type of surgery (*p* = 0.531), and perioperative radiation therapy (*p* = 0.202) between the TM-ILP and standard groups (Table 2).

**Table 2:** Treatment characteristics

	Non-ILP	ILP	*p*
Resectability			0.008
Resectable	9 (17%)	2 (4%)	
Locally advanced—non-resectable	15 (29%)	26 (50%)	
Surgery			0.531
Limb-sparing surgery	20 (39%)	25 (48%)	
Amputation	4 (8%)	3 (6%)	
Resection margins			0.898
Negative			
R0	15 (29%)	16 (31%)	
Positive			
R1	8 (15%)	11 (21%)	
R2	1 (2%)	1 (2%)	
Radiotherapy			0.202
Neoadjuvant	4 (7.7%)	5 (9.6%)	
Adjuvant	6 (11.5%)	2 (3.8%)	
No	14 (27%)	21 (40.4%)	

### Subsequent recurrence-free survival

**Fig. 1:**
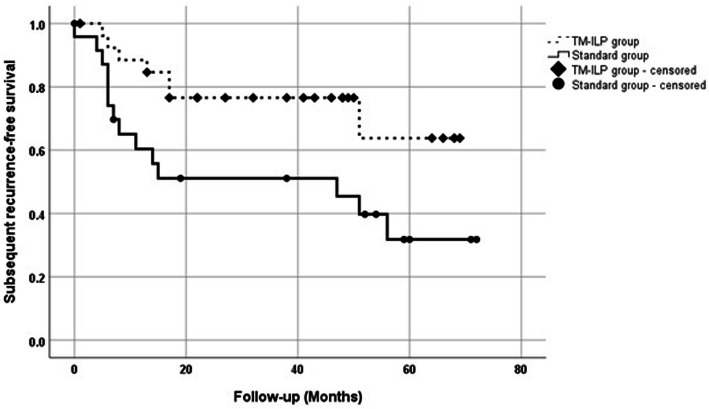
Kaplan-Meier plot of subsequent recurrence-free survival of patients in TM-ILP and standard groups (52 cases from 47 patients)

**Fig. 2:**
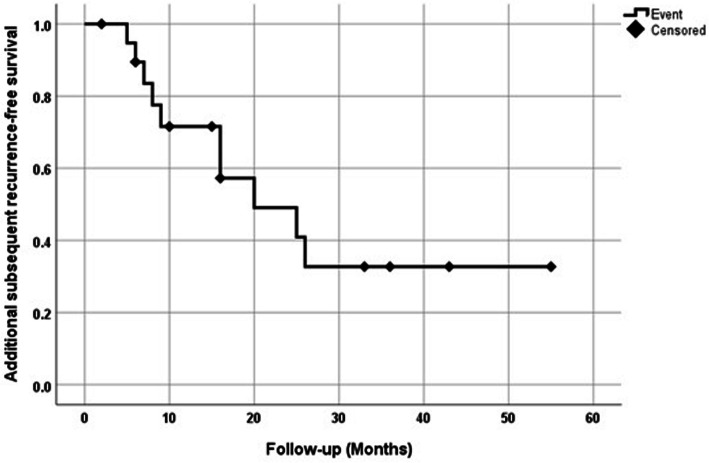
Kaplan-Meier plot of recurrence-free survival of patients with additional subsequent local recurrences (*N* = 20 patients)

At the time of analysis, median follow-up for SRFS was 45 months (interquartile range (IQR) 23–56). Subsequent local recurrences were observed in 20 (39%) patients, including 6 (30%) in the TM-ILP-group and 14 (70%) in the standard group. Three-year RFS for the TM-ILP-group was 75% (CI 95% 71.5–78.5) and differed significantly (*p* = 0.026) from the standard group 43.4% (CI 95% 38.7–48.1) (Fig. 1). Out of six patients with subsequent local recurrences in TM-ILP-group in five patients, it was pathological NC and by one patient CR in tumor specimen after TM-ILP. Additional surgery treated 18 of these recurrences, while two others were treated with palliative chemotherapy only, due to multiple distant metastases (Table 3). The median time of additional subsequent recurrence was 15 months (IQR, 6–25). The 12-month survival for additional subsequent recurrence was estimated at 68% (CI 95% 62.9–73.1; see Fig. 2).

**Table 3:** Multivariable Cox regression analysis of prognostic factors for subsequent local recurrence in 52 cases (backward stepwise selection)

	HR (95% CI)	*p*
Resection margins		0.036
Negative ^a^		
Positive	2.63 (1.06–6.5)	
ILP		0.036
Yes ^a^		
No	2.6 (1.06–6.6)	
Number of recurrences		0.005
Subsequent local recurrences	3.96 (1.5–10.5)	

^a^ reference group for proportional hazard ratio regression

Abbreviations: *HR* hazard ratio, *CI* confidence interval, *p p* value, *a* reference group for proportional hazard ratio regression

### Distant metastasis-free survival

**Fig. 3:**
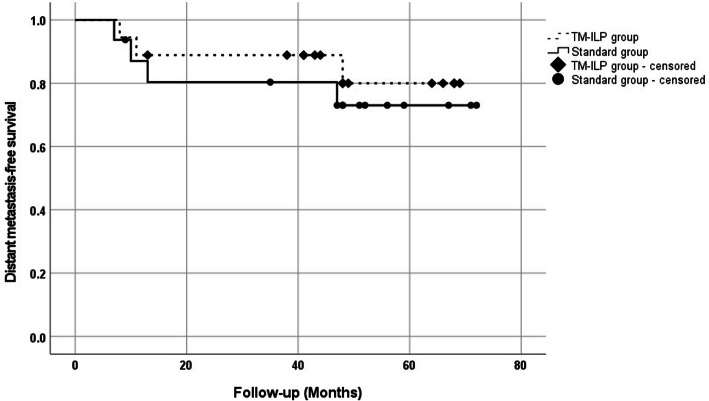
Kaplan-Meier plot of disease-specific survival of patients in TM-ILP and standard groups (*N* = 35 patients)

The univariate Kaplan-Meier disease-specific survival analysis for both TM-ILP and non-TM-ILP groups included 35 patients (67%) who had no metastasis at the time of local recurrence diagnosis. The median follow-up was 44 months (IQR 20–100). the distant metastasis-free survival of 18 patients in the TM-ILP-group (44 months: OS = 88.9%; 95% CI (85.2–92.6%) was the absence of any significant evidence (*p* = 0.572) in comparison to the standard group with 17 patients (44 months OS = 80.4%; 95% CI (75.2–85.6)) (Fig. 3).

### Multivariable analysis of local recurrences

Backwards and forwards stepwise selection identified positive resection margins (*p* = 0.036), TM-ILP (*p* = 0.036), and treatment of the second or additional local recurrences (*p* = 0.005) as an independent risk factor for the development of local recurrence.

The absence of TM-ILP (hazard ratio (HR) 2.6, 95% CI (1.06–6.6)) and positive resection margins (HR 2.63, 95% CI (1.06–6.5)) were associated with less favorable outcomes. Furthermore, patients resected for a first local recurrence were nearly four times less likely (HR 3.96, 95% CI (1.5–10.5)) to develop subsequent local relapse (Table 3).

## Discussion

The local recurrence in high-grade soft tissue sarcoma occurs in 20–30% of all cases [15, 16]. Due to previous surgery and radiation therapy, the management of locally recurrent high-grade STS is often considerably more difficult than that of primary STS. Thus, treatment outcomes of recurrent STS are associated with significantly greater morbidity and a less favorable functional outcome [17, 18]. This novel study sought to exclusively compare locally recurrent soft tissue extremity sarcoma treated by TM-ILP and subsequent resection of the residual tumor to a group of comparable tumors treated by standard surgical therapy without the use of TM-ILP.

The decision to implement TM-ILP was determined by an interdisciplinary tumor-board and was mainly contingent on localization and involvement of critical neurovascular structures. In order to reduce a potential selection bias, all patients with tumors extending proximally above the axilla or the groin were not included in this study, because in these cases, an isolation of the tumor during TM-ILP would not have been possible.

Because this study did not randomize patients into treatment groups, multimorbid patients and patients with resectable tumors were more likely to receive the standard (non-TM-ILP) treatment. Consequently, we expected a higher number of resectable tumors in the standard treatment group; thus, one would have expected a positive selection bias in terms of a potentially better starting position, a better resectability, and less risk of local recurrence in this treatment group. Most surprisingly, we observed quite the opposite: the local recurrence-free survival was significantly higher in the TM-ILP group compared to the standard group. In addition, the demographic and tumor-specific data showed a comparability of both groups with regard to further potential influencing factors: previous radiation therapy, number of local recurrences prior to treatment, tumor size, and pathohistological subtypes showed no significant differences.

These findings suggest a potential benefit from the implementation of TM-ILP into a multimodal treatment regimen. In a retrospective analysis of 62 patients with locally recurrent STS, Torres et al. could show that additional radiation therapy did not improve recurrence-free survival compared to surgical excision alone with local control rates of 50% in 5 years [19]. On the other hand, repeated radiation therapy can result in severe functional problems of the extremity. Often the cumulative dose can exceed 100 radiation dose in Grey, which can lead to soft tissue necrosis, rupture of vessels, or malperfusion of the extremity, neuropathy of major peripheral nerves and osteonecrosis [19–21]. These severe complications can occur in more than 50% of patients treated with repeated radiation therapy. Even though plastic reconstructive surgery may solve some of these problems [22], many authors recommend amputation rather than retreatment by radiotherapy [23–26]. In contrast, TM-ILP can be applied repeatedly without an increased risk of complications or increased local toxicity [27, 28].

Several previous studies described an estimated risk of up to 30% for locally recurrent STS when surgical resection resulted in positive resection margins; this was especially true in large, high-grade tumors [16, 29, 30]. We confirmed these observations in our proportional hazards analysis which showed that resection of local recurrences with positive resection margins were three-times more likely to develop a subsequent local recurrence than those with negative margins.

The development of a first local recurrence has in itself been shown to be associated with increased risk of a subsequent local relapse [30]. Our multivariate study could confirm these findings; a first local recurrence treated adequately has a higher chance of achieving a state of permanent local tumor control than a subsequent local recurrence. This emphasizes the importance of aggressive and intense multimodal treatment at this stage.

As anticipated, distant metastasis-free survival was similar in both groups, which again confirms the postulate that adequately performed local therapy does not influence disease-specific survival in STS [31–33].

The limitations of this study are primarily its retrospective character, the rather small patient cohorts, and the fact that patient allocation to treatment groups could not be randomized. The latter has already been extensively discussed in the TM-ILP-community by Gronchi and Bonvalot: “ILP and RT: the study that will never be” [34]; therefore, we are especially pleased to be able to refute this statement and contribute novel data regarding this issue. Despite the previously mentioned limitations, we argue the importance of this study; it presents a novel direct comparison of patients treated with TM-ILP to a group of patients treated without TM-ILP. We do concede that a possible selection bias would lead to a potentially better initial position in the standard group. Despite this fact, we observed a local control rate that was still significantly higher in patients who were treated by TM-ILP and subsequent tumor resection. Hence, we maintain that this study provides compelling evidence for the treatment of locally recurrent extremity STS with the support of TM-ILP. Nevertheless, we agree that further analysis of this matter is necessary, preferably in a prospective and randomized setting.

In conclusion, this study revealed the importance of TM-ILP for the treatment of locally recurrent soft tissue sarcoma of the extremities. Implementing TM-ILP prior to resection of the recurrent tumor appears to effectively lower the risk of subsequent local recurrence; further analysis is advisable to confirm these findings.

## Data Availability

The data that support the findings of this study are available from the corresponding author (Farhad Farzaliyev, MD) on request.
